# A Role of BDNF in the Depression Pathogenesis and a Potential Target as Antidepressant: The Modulator of Stress Sensitivity “Shati/Nat8l-BDNF System” in the Dorsal Striatum

**DOI:** 10.3390/ph14090889

**Published:** 2021-09-01

**Authors:** Hajime Miyanishi, Atsumi Nitta

**Affiliations:** Department of Pharmaceutical Therapy and Neuropharmacology, Faculty of Pharmaceutical Sciences, University of Toyama, 2630 Sugitani, Toyama-shi, Toyama 930-0194, Japan; d2062305@ems.u-toyama.ac.jp

**Keywords:** BDNF, dorsal striatum, stress sensitivity, depression, Shati/Nat8l

## Abstract

Depression is one of the most common mental diseases, with increasing numbers of patients globally each year. In addition, approximately 30% of patients with depression are resistant to any treatment and do not show an expected response to first-line antidepressant drugs. Therefore, novel antidepressant agents and strategies are required. Although depression is triggered by post-birth stress, while some individuals show the pathology of depression, others remain resilient. The molecular mechanisms underlying stress sensitivity remain unknown. Brain-derived neurotrophic factor (BDNF) has both pro- and anti-depressant effects, dependent on brain region. Considering the strong region-specific contribution of BDNF to depression pathogenesis, the regulation of BDNF in the whole brain is not a beneficial strategy for the treatment of depression. We reviewed a novel finding of BDNF function in the dorsal striatum, which induces vulnerability to social stress, in addition to recent research progress regarding the brain regional functions of BDNF, including the prefrontal cortex, hippocampus, and nucleus accumbens. Striatal BDNF is regulated by Shati/Nat8l, an *N*-acetyltransferase through epigenetic regulation. Targeting of Shati/Nat8l would allow BDNF to be striatum-specifically regulated, and the striatal Shati/Nat8l-BDNF pathway could be a promising novel therapeutic agent for the treatment of depression by modulating sensitivity to stress.

## 1. Introduction

In recent years, the number of patients with various psychiatric diseases, such as mood disorders, including major depressive disorder (depression) and bipolar disorder diagnosed by the Diagnostic and Statistical Manual of Mental Disorders-5 (DSM-5) [1], has been increasing. Among these disorders, depression is one of the most serious and common. The World Health Organization (WHO) reported more than 300 million patients with depressive symptoms in 2018 [2]. The prevalence of depression in lifetime reached 15–18% [3], meaning that one in five people were affected by the condition in their lifetime. The symptoms of depression include deficits in cognitive function [4,5], loss of motivation [6], and anhedonia [7]. Furthermore, depression is strongly associated with a risk of low energy and the presence of suicidal ideation [8,9]. Considering these symptoms, depression greatly affects the lives of its sufferers. In the last 50 years, serotonin has been recognized as a therapeutic target for depression. Consequently, tricyclic antidepressants have been developed to control the amount of serotonin in the brain [10]. Currently, selective serotonin reuptake inhibitors (SSRIs) or serotonin and noradrenaline reuptake inhibitors (SNRIs) are used as first-line therapeutic tools for depression. However, the use of these antidepressant therapies is limited. Generally, these drugs require long periods before the effects are seen [11] and do not show high response rates in approximately 30% of patients with depression, who are resistant to treatment [12,13]. Furthermore, their side effect occurs frequently in depression patients with antidepressant treatment [14]. Indeed, the United States Food and Drug Administration (USFDA) warned of treatment-associated suicidality as an adverse event associated with antidepressant treatment [15]. Thus, in the last decade, there has been an increased focus on the discovery of novel targets for depression therapy and the establishment of treatment strategies that work for all patients, including those with treatment resistance. However, there are little progress for the providing the attractive treatment to patients with treatment resistance.

Stress is closely related to the pathogenesis of depression [16]. Studies have shown that depression onset is caused by stressful environments [17,18,19]. Depressive events such as stress induce vulnerability to depression onset; moreover, subsequent even mild stressful events can trigger depression onset, depending on the level of vulnerability to stress [20,21,22]. However, not all individuals are vulnerable to depression onset, as some remain resilient [23,24,25]. Rodents exposed to repeated social-defeat stress, which has been used as a major stressful event, can also be classified as either stress-susceptible (showing depression-like behaviors) or resilient (lack of depression-like behaviors) [26,27] However, the underlying molecular mechanisms that contribute to resilience against stress remain poorly understood. Clarifying the regulatory mechanisms of stress sensitivity could provide novel insights for understanding the pathogenesis of depression, and modulating stress sensitivity reduces damage from stress and accelerates the recovery as a novel strategy for depression.

One possible factor triggering depression is the involvement of brain-derived neurotrophic factor (BDNF) mediating significant atrophy and structural changes in the brain, as part of the BDNF-hypothesis of depression. As alterations in BDNF levels directly affect the pathogenesis of depression, the regulation of these levels might lead to the development of treatments for depression [28]. The role of BDNF in the central nervous system (CNS) in depression may depend on the circuit and region, with both antidepressant and antidepressant effects [29,30]. Recently, BDNF in the dorsal striatum was reported to regulate the stress sensitivity.

In this review article, we first provide the background of the present situation and recent research progress regarding the brain regional functions of BDNF, including the prefrontal cortex (PFC), hippocampus, and nucleus accumbens (NAc), in the pathophysiology of depression. We also highlighted BDNF function in the dorsal striatum as a novel role in depression as a regulator of stress sensitivity. Finally, we discussed how to regulate BDNF dorsal striatum-specifically and the application of these findings as a novel strategy for the treatment of depression.

## 2. The Role of Brain-Derived Neurotrophic Factor in the Pathology of Depression

Nerve growth factor (NGF) was identified in the early 1950s as the first member of the neurotrophin family, which induced neuron growth and survival promotion [31]. In 1982, BDNF was discovered and purified from the pig brain as the second member of the neurotrophin family that also includes neurotrophin-3 and neurotrophin-4 and which facilitates neuronal survival in the dorsal root ganglion [32]. BDNF is the most abundantly expressed member of the neurotrophin family in the CNS [33] and it plays an important role in neuronal development, morphology, and synaptic formation and function in the whole brain [34]. Very few BDNF knockout mice reach adulthood; those that do so show severe sensory impairment [35]. BDNF also affects learning and memory, as well as neuronal survival, through the regulation of synaptic plasticity [36,37].

BDNF has a high affinity for the tropomyosin receptor kinase B (TrkB) receptor, a member of the receptor tyrosine kinase family. TrkB receptors exist both pre- and post- synapse [38]. The phosphorylation of TrkB induced by the binding of BDNF to the TrkB receptor can regulate at least three intracellular cascades; namely, phospholipase C-γ (PLC-γ), Ras-mitogen-activated protein (MAP) kinase/extracellular signal-related kinase (MAPK/ERK), and phosphatidylinositol 3-kinase/protein kinase B (PI3K/AKT) signaling [39,40,41].

The BDNF gene has nine promoters, each of which drives the expression of distinct BDNF transcripts [42,43]. The contribution of the region-specific BDNF effect is attributed to the distinct BDNF transcripts [44]. The promoter of BDNF IV is the most well-characterized promoter that responds to stress in depression pathogenesis, as rodent models of depression exposed to social-defeat stress showed decreased mRNA levels of total BDNF and BDNF IV but neither BDNF transcripts [45] in response to restraint stress. These findings are consistent with those of a clinical study in which patients with depression also showed reduced BDNF IV expression in the hippocampus and frontal cortex, as well as in animal models of depression [46,47].

Alteration of BDNF protein levels in both the brain and serum have been observed in patients with brain illnesses [48,49,50]. BDNF is also involved in psychiatric diseases such as schizophrenia, autism, and depression [51,52,53]. As mentioned previously, the function of BDNF in depression pathogenesis is heterogeneous, depending on the brain region and individual circuits. BDNF shows an antidepressant function in the PFC [54,55] and hippocampus [56,57] and a pro-depressant function in the BDNF in the mesolimbic dopamine (DA) circuit, originating in ventral tegmental area (VTA) DA neurons that project to the NAc [58]. We next review the roles of BDNF in these regions, as well as the involvement of the dorsal striatum in depression pathogenesis, with a focus on stress sensitivity.

### 2.1. Prefrontal Cortex

The PFC plays an important role in learning, memory, and decision-making processes [59,60]. The PFC is also involved in the pathogenesis of depression. Changes in structures and function in the PFC caused by altered dysfunction of the neuronal circuit have been reported in human studies. The methods for the treatment of depression treatment include electrical stimulation, including transcranial direct-current stimulation (tDCS) [61] and electroconvulsive therapy (ECT) [62], or magnetic stimulation including magnetic seizure therapy (MST) [63] and repetitive transcranial magnetic stimulation (rTMS) [64] to transiently induce neuronal activity. Deep brain stimulation (DBS) also enables the invasive region-specific regulation of neuronal activity using implanted electrodes [65]. All of these treatments normalize neuronal activity in the PFC and have been shown to have antidepressant effects [66]. A rapid-acting antidepressant agent, ketamine, an *N*-methyl-*D*-aspartate receptor (NMDAR) antagonist, also transiently activates the PFC neuron [67], with an antidepressant effect in patients with depression, even those with treatment resistance [68].

Clinical studies of postmortem brains have reported decreased BDNF and TrkB levels in the PFC of subjects who committed suicide [69,70] and patients with depression [71,72]. These subjects also showed atrophy of neurons in the PFC [73], suggesting that downregulation of BDNF signaling was strongly associated with atrophy of the PFC in patients with depression.

The causal relationships between neuronal morphology in the PFC, alteration of BDNF expression in the PFC, and depression-like behaviors have been investigated in rodent models of depression exposed to stressors including repeated social-defeat stress (RSDS), chronic unpredictable stress, and chronic restraint stress. The rodents exhibited depression-like behavior due to these chronic stresses, as well as decreased numbers of spines and branches and length of dendrites in the PFC [74,75]. These findings indicated that chronic stress induced neuronal atrophy in the PFC of rodents, consistent with the results of the postmortem studies described above. Chronic stress decreases levels of BDNF and phosphorylated TrkB in the PFC, indicating the inactivation of TrkB signaling [76,77]. BDNF mutant knock-in mice exhibited decreased length and branching of apical dendrites in PFC neurons and vulnerability to chronic stress [78,79]. These results suggested that BDNF in the PFC contributes to the etiology of depression through neuronal atrophy. This supposition is supported by evidence that inhibition of TrkB signaling using ANA-12, a TrkB antagonist, restored depression-like behaviors with the recovery of dendrite abnormalities in animal models [80]. These findings are consistent with the results that microinfusion of BDNF into the PFC has antidepressant effects [81].

Many studies have focused on the PFC as the region displaying altered function, potentially triggered by altered glutamatergic and gamma-aminobutyric acid (GABA) transmission [82]. The acute antidepressant effect of ketamine has also been reported to be involved in the PFC [83]. Preclinical studies have revealed that ketamine treatment rapidly induced glutamate release in the PFC via regulation of synaptic plasticity [73]. This enhancement of glutamate release is caused by the inhibition of NMDARs on GABAergic interneurons that downregulate glutamate transmission [83]. Increased glutamate release then stimulates postsynaptic AMPARs, and L-type voltage-dependent calcium channels are depolarized and activated, followed by BDNF [84,85]. The antidepressant effect of ketamine disappeared in BDNF conditional knockout mice; moreover, microinfusion of an anti-BDNF neutralizing antibody into the mPFC also suppressed this antidepressant effect [81].

Taken together, these findings indicate that BDNF in the PFC plays an important role in the antidepressant effect and is essential for the use of ketamine as an antidepressant.

### 2.2. Hippocampus

The hippocampus controls memory and learning and emotional processing and is susceptible to the effects of stress [86]. Neuronal plasticity in the hippocampus is altered by stress, which increases the risk of depression [87].

We would like to introduce a clinical study examining a BDNF polymorphism (Val66Met) in depression. The variant is involved in the onset of depression, anxiety, and increased suicidal ideation and has been observed in the hippocampus of patients with depression [88,89,90]. The presence of the BDNF Val66Met polymorphism impairs BDNF release [91] and the maturation of BDNF from proBDNF in the hippocampus [92]. Smaller hippocampal volumes have also been reported in BDNF Val66Met-variant carriers [93,94], suggesting a genetic association between the BDNF Met variant, hippocampal shrinkage, and the risk of depression.

Neurogenesis in the hippocampus contributes to antidepressant effects [95,96]. While impaired hippocampal neurogenesis can lead to depression [97], BDNF upregulation stimulates hippocampal neurogenesis, followed by an induced antidepressant effect [98]. Several studies have demonstrated the relationship between BDNF and morphological changes caused by hippocampal neurogenesis dysfunction in patients with depression. Structural alterations, including decreased hippocampal and PFC volumes, have been reported in patients with depression [99,100]. In addition, postmortem examinations showed reduced BDNF protein levels in the hippocampus of patients with depression patients; however, individuals receiving antidepressant treatment did not show a reduction in BDNF protein levels in the same regions [101]. Similar results were also observed in rodent experiments [102]. Decreased BDNF levels and reduced numbers and density of dendritic spines were observed in a mouse model of depression exposed to RSDS [103]. In addition, hippocampal BDNF knockdown mice, microinjected with lentiviral vectors, showed reduced neurogenesis and depression-like behaviors [104]. Treatment with an antidepressant increased BDNF expression and restored the impairments of the dendritic spine [105]. Similarly, microinfusion of BDNF into the hippocampus restored depression-like behaviors induced by chronic stress [106].

A loss of serotonergic neurons was observed in a mouse model of depression due to reduced BDNF in the hippocampus, since these neurons grow and survive via BDNF-TrkB signaling cascades [107]. Antidepressant medications also activate TrkB receptors [108], suggesting the involvement of serotonergic neurotransmission in the enhancement of BDNF-TrkB signaling cascades. Interestingly, the conditional overexpression of BDNF in the hippocampus also showed an antidepressant effect even in serotonin transporter knockout rats, which showed depression-like behaviors [109]. The results of these postmortem and animal studies showed that BDNF in the hippocampus was involved in the serotonergic system and depression pathogenesis, indicating that normalizing BDNF signal activity, followed by restoration of hippocampal atrophy and normalization of impaired serotonergic transmission, might be a novel approach to improving the symptoms of depression.

Taken together, these findings suggest that decreased BDNF levels in the hippocampus are tightly linked to morphological alterations and depression pathogenesis in patients.

### 2.3. Mesolimbic Pathway

Mesolimbic BDNF function is also reportedly involved in the pathogenesis of depression [110]. In contrast to the antidepressant effect of BDNF in the PFC or hippocampus, BDNF signaling in the mesolimbic regions has the opposite, pro-depressant, effect. A clinical study showed increased BDNF protein levels in the NAc in patients with depression [111]. Furthermore, the reduction of BDNF expression in the ventral tegmental area (VTA) by electroconvulsive therapy has an antidepressant-like effect [112]. In animal experiments, chronic stress, including chronic unpredictable mild stress and RSDS, induced increased BDNF protein levels and activation of BDNF-TrkB signaling [29]. Ablation of BDNF in the NAc induced an antidepressant effect in the social defeat session [29]; the inhibition of BDNF-TrkB signaling in the NAc with a truncated mutant with TrkB overexpression also showed the same results [113]. In addition, mice with BNDF knockout in the VTA showed resilience to RSDS and induced anti-depressant-like effects [29]. These results suggest that BDNF in the mesolimbic regions, especially the VTA–NAc circuits, are involved in the pathophysiology of depression and have antidepressant effects.

BDNF and TrkB are expressed in the mesolimbic dopamine (DA) circuit, which projects from the VTA to the NAc [114,115]. This mesolimbic dopaminergic system was first considered to contribute to drug addiction [116]. Recent studies have provided evidence of an association between the mesolimbic dopaminergic system and motivation- and mood-related behaviors, including social interaction [117,118]. Considering the loss of happiness and motivation as the main symptoms in patients with depression [119], it is quite likely that abnormalities of the mesolimbic DA system are involved in depressive-like outcomes [58,120]. This circuit is activated by some chronic stress; importantly, the activation of DA neurons was observed in stress-susceptible but not stress- resilient mice [110]. Optogenetic activation of the VTA to NAc DA neurons rapidly induced depression-like phenotypes in mice previously classified as resilient that were exposed to RSDS and also showed vulnerability to RSDS, and even subthreshold social-defeat stress (microdefeat stress) [121]. In contrast, optogenetic inhibition of the VTA in NAc DA neurons induced resilience to social stress. In the VTA-to-PFC circuits, decreased and increased firing activities were observed in the susceptible and resilient groups, respectively [121], suggesting a region-specific function of DA neurons in depression pathogenesis.

Notably, we emphasized that BDNF-TrKB but not DA signaling in the VTA–NAc circuits is important for the development of depression-like behaviors. While microinfusion of the TrkB antagonist, ANA-12, into the NAc inhibited the induction of decreased social ability in RSDS, microinfusion of a DA receptor antagonist did not have this effect [122]. In addition, the vulnerability to social stress induced by optogenetic activation of the VTA–NAc circuits disappeared with the inhibition of BDNF-TrkB signaling in the NAc by a TrkB antagonist [122], suggesting that increased BDNF release, rather than DA, in the NAc is essential for the development of depression pathology.

Taken together, these findings suggest the pro-depressant effect of BDNF in the mesolimbic region, especially the VTA–NAc pathway.

### 2.4. Dorsal Striatum

The dorsal striatum is classically described as playing a key role in motor function. The dorsal striatum is a part of the basal ganglia, which is important for the adjustment of the execution of motor habits [123]; thus, deficits in motor automaticity are a characteristic of basal ganglia-related illnesses, such as Parkinson’s disease [124]. The striatum mainly receives direct excitatory input from the primary motor and sensory cortices [125], suggesting the involvement of the striatum and motor function. Recent studies have also reported that the striatum also plays an important role in the reward and learning systems, especially in decision-making [126,127,128]. Initiation and choice of action and motivational and emotional behaviors are regulated by specific dorsal–striatal circuits [129]. Lesions in the dorsal striatum affect the establishment of habitual behaviors [130] and inactivation of the dorsal striatum weakens the reward system and induces goal-directed action even in the absence of a reward [131,132]. Thus, the dorsal striatum plays an essential role in decision-making, learning, and reward systems.

Although there are no reports on the relationship between the dorsal striatum and depression, anxiety, which is one of the main symptoms of depression, may be regulated by the dorsal striatum [133]. As the striatum-related circuit mechanisms underlying anxiety, the striatal-prefrontal pathway is involved and becomes less connected, the cortico-striatal connections are also impaired, and anxiety is expressed [134].

Our previous study was the first to report the role of the dorsal striatum in the pathogenesis of depression, except for anxiety [135]. We recently showed BDNF function in the dorsal striatum of patients with depression. We found that stress-susceptible but not stress-resilient mice showed increased *BDNF* mRNA and protein levels after RSDS compared to stress-naïve mice [136]. The reported hypertrophy of the dorsal striatum in response to chronic stress is supported by our results showing increased BDNF in the dorsal striatum by RSDS, as BDNF is required for neurogenesis [137]. We also revealed that the expression levels of BDNF mRNA are correlated with social interaction behaviors [136]. To investigate whether BDNF in the dorsal striatum induced depression-like behaviors, ANA-12 was microinfused into the dorsal striatum 15 min before each social-defeat stress session for 10 days. ANA-12 treated mice showed stress resilience to RSDS in all behavioral experiments, including social-interaction, sucrose-preference, tail-suspension, and forced-swimming tests [136]. These results suggested that BDNF-TrkB signaling in the dorsal striatum regulates stress sensitivity. Notably, an antidepressant effect was not observed in stress-naive mice infused with ANA-12 into the dorsal striatum, indicating that striatal BDNF reflects sensitivity to stress and not to depression-like behaviors.

A recent study proposed a double-hit hypothesis for the onset of depression. This hypothesis is composed of two combined factors of genetic and environmental elements in the development of a psychiatric illness, including depression [138,139]. Vulnerability to the onset of depression is caused by a hereditary factor (first hit; abnormal depression-related genes), followed by an environmental factor (second hit; life stress) to induce the onset of the pathogenesis. The RSDS method is used as the environmental factor (second hit) and classify two groups of mice as vulnerable to the onset of depression or stress resilience. The results of our study show that vulnerability to social stress in mice, elevated dorsal striatal BDNF mRNA levels, and local inhibition of BDNF-TrkB signaling induced resilience to social stress [136], suggesting that BDNF in the dorsal striatum is involved in the sensitivity to social stress as a hereditary factor related to the onset of depression. Although BDNF expression requires confirmation in future human studies, these results support the double-hit hypothesis and provide a novel aspect of stress sensitivity in depression pathogenesis.

**Figure 1:**
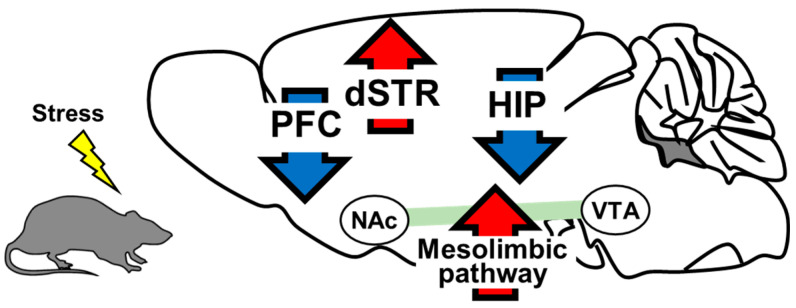
BDNF levels in rodent model of depression. BDNF expression in the PFC and hippocampus was decreased in the RSDS-induced depression model mice. BDNF in the PFC and hippocampus has an anti-depressant effect. BDNF expression in the NAc and dorsal striatum (dSTR) increased in a repeat social-defeat stress (RSDS)-induced depression mouse model. BDNF in the NAc and dorsal striatum shows a pro-depressant effect. Red arrows: upregulation of BDNF levels. Blue arrows: downregulation of BDNF levels.

At the end of this section, we summarize the alteration of BDNF levels in the brain, including PFC, hippocampus, NAc, and dorsal striatum in depression individuals in Figure 1 and Table 1.

## 3. Shati/Nat8l

We demonstrated that Shati/Nat8l, a mental disorder-related gene, is upstream of BDNF in the dorsal striatum and upregulates striatal BDNF expression by promoting epigenetic modification.

Shati/Nat8l was previously identified in the NAc of a mouse model of psychosis administered repeated methamphetamine treatment [140]. Shati/Nat8l was later recognized to have N-acetyl transfer activity and is a protein that synthesizes *N*-acetylaspartate (NAA) from acetyl-CoA and aspartate [141]. NAA is distributed at high concentrations in the CNS, and is biosynthesized to *N*-acetylaspartylglutamate (NAAG) by condensation with glutamic acid [142]. NAAG is widely present in the brains of mammals [143] and functions as a highly selective neurotransmitter for group II metabotropic glutamate receptor 3 (mGluR3) [144]. NAAG is metabolized to NAA and glutamate by glutamate carboxypeptidase II (GCPII) [145], which are then metabolized to aspartate and acetylate and finally converted to acetyl-CoA by aspartoacylase (ASPA) [146]. NAA levels are altered in the brains of patients with mental disorders, including depression, schizophrenia, and bipolar disorder [147].

Shati/Nat8l and NAA have various functions in the CNS. We show that Shati/Nat8l in the PFC was related to the reward system. Shati/Nat8l overexpression in mouse PFCs (PFC-Shati OE mice) was generated using an adeno-associated virus (AAV) vector. An in vivo microdialysis study showed reduced extracellular DA levels and suppression of the methamphetamine-induced elevation of DA in the NAc of these mice. These mice also showed attenuation of METH-induced conditioned place preference (CPP); however, locomotor activity was not changed [148,149].

Learning and memory are also involved in Shati/Nat8l expression in the hippocampus. Shati/Nat8l overexpression in the mouse hippocampus (HIP-Shati OE mice) was generated using an AAV vector; these mice were then subjected to learning and memory tests. In the novel object test, the HIP-Shati OE mice preferred to approach the novel object in the trial session compared with control mock mice [150].

### Shati/Nat8l and Depression

A recent study in our laboratory provides a deep understanding of the crucial role of Shati/Nat8l in depression pathogenesis and stress sensitivity, in addition to previous findings on the function of Shati/Nat8l.

A previous human study showed that Shati/Nat8l was a diagnostic biomarker for depression [151]. Miyamoto et al. demonstrated increased Shati/Nat8l in the dorsal striatum following repeated forced-swimming stress [135]. Uno et al. also demonstrated upregulated striatal Shati/Nat8l expression by RSDS [152]. The expression of Shati/Nat8l in regions other than the dorsal striatum was not altered in these studies. Furthermore, the elevation of Shati/Nat8l by chronic stress was observed in stress-susceptible, non-resilient mice [136]. To investigate the role of Shati/Nat8l in the dorsal striatum in depression, striatal Shati/Nat8l overexpression (STR-Shati OE mice) and knockdown (STR-Shati cKD mice) mice were generated. The STR-Shati OE mice were vulnerable to social-defeat stress. The ratio of stress-susceptible mice after RSDS increased three-fold with Shati/Nat8l overexpression compared with control mock mice. Furthermore, these mice also showed susceptibility to microdefeat stress [152]. Decreased serotonin levels were observed in the brains of STR-Shati OE mice [135]. The impairment of stress sensitivity in these mice was recovered by treatment with the selective serotonin reuptake inhibitor (SSRI), fluvoxamine [152], suggesting that the serotonergic system is downstream of the Shati/Nat8l pathway in depression pathogenesis. In contrast, resilience to RSDS was established by conditional knockdown of Shati/Nat8l in the dorsal striatum. Unlike in the STR-Shati OE mice, the ratio of stress-resilient mice increased in STR-Shati cKD mice. In addition, the Shati cKD mice did not show depression-like behaviors even after strong RSDS, while almost all control mock mice showed depression-like behaviors after this stress [136]. We also found increased BDNF and Shati/Nat8l levels in the dorsal striatum in the stress-susceptible but not stress-resilient mice, as well following RSDS. The expression of dorsal striatal BDNF was correlated with the expression of Shati/Nat8l in the dorsal striatum, and BDNF protein levels were suppressed in the Shati cKD mice, suggesting that BDNF in the dorsal striatum is downstream of Shati/Nat8l [136]. This result supports the alteration of BDNF mRNA levels in some brain regions of genetic Shati/Nat8l KO mice [153].

To strengthen the evidence of BDNF regulation by Shati/Nat8l in the dorsal striatum, we investigated BDNF levels in STR-Shati OE mice. BDNF mRNA levels in the dorsal striatum were shown to increase by overexpression of dorsal striatal Shati/Nat8l compared to those in mock mice (Miyanishi et al., unpublished data). Considering the function of dorsal striatal BDNF in stress sensitivity [136], the Shati/Nat8l-BDNF pathway in the dorsal striatum determines resilience or vulnerability to stress.

As mentioned above, before our previous study, no other study had demonstrated the role of dorsal striatal function in stress sensitivity. Many studies have shown that networks control the functions of emotional behaviors, motivation, and negative decision-making with other brain regions [133,154]. The contribution of the dorsal raphe nucleus (DRN) as a modulator of stress sensitivity among networks within the dorsal striatum is considerable. Neurons in the DRN are abundant in serotonin and a lack of serotonin is strongly involved in depression. The hereditary reduction of serotonin in the brain induced vulnerability to social stress in mice [155] and the replenishment of serotonin by SSRI is used as the first-line treatment for depression. These considerations are consistent with our report that dorsal striatal overexpression of Shati/Nat8l induced vulnerability to stress accompanied by a decline in serotonin [135]. The impairment of sensitivity to stress by the overexpression of Shati/Nat8l in the dorsal striatum was attenuated by activation of DRN or systemic administration of SSRI [152]. These findings suggested that Shati/Nat8l overexpression in the dorsal striatum inactivates serotonergic systems from the DRN, thereby suppressing serotonin release. In this context, the stress resilience observed in STR-Shati cKD mice was probably induced by the activation of serotonergic neurons from the DRN. As the Shati/Nat8l-BDNF pathways are involved in sensitivity to stress, these results suggest that the serotonergic system from the DRN could be downstream of the Shati/Nat8l-BDNF pathways in the dorsal striatum, indicating that the regulation of serotonin release in the brain by dorsal striatal Shati/Nat8l-BDNF pathways determines stress sensitivity.

One possible mechanism of BDNF regulation in the dorsal striatum by Shati/Nat8l is epigenetic regulation. Previous studies have reported that BDNF in the brain is regulated by epigenetic mechanisms, including histone methylation and acetylation and DNA methylation. Histone methylation and acetylation are generally considered to inhibit and activate gene transcription, respectively [156,157]. DNA methylation is also a major epigenetic modification that regulates gene expression [158]. Alterations in histone methylation in the promoter region of BDNF by stress were associated with depression-like behaviors in mice [159]. BDNF production was altered by stress via histone acetylation, which then induced depression-like behaviors in mice [46]. DNA methylation levels in the promoter region of BDNF also increased after stress exposure [160]. These findings suggested that epigenetic modifications regulate BDNF and play an important role in depression. In the dorsal striatum, we found increased acetylation levels at histone H3K9, but not histone and DNA methylation, in the BDNF promoter regions in stress-susceptible but not stress-resilient mice [136]. This result is consistent with the upregulation of dorsal striatal BDNF expression in stress-susceptible mice because histone acetylation generally promotes gene transcription. In addition, decreased H3K9ac levels were observed in Shati cKD mice, indicating stress resilience. By performing chromatin immunoprecipitation assay, in contrast, it is showed that overexpression of Shati/Nat8l in the dorsal striatum induced histone H3K9 acetylation (Miyanishi et al., unpublished data). These results indicate that dorsal striatal BDNF expression is regulated by Shati/Nat8l, which mediates histone H3K9 acetylation. Shati/Nat8l predominantly produces NAA [161], which is ultimately converted to acetyl-CoA [146]. Elevation of NAA production via the upregulation of Shati/Nat8l after RSDS in susceptible mice, followed by an increase in acetyl-CoA, a substrate of histone acetylation, might cause enhanced H3K9acetylation. NAA levels in the dorsal striatum were increased in STR-Shati OE mice [152], which were vulnerable to social stress, consistent with our hypothesis.

To summarized this section, BDNF expression in the dorsal striatum, not but other regions, is regulated by Shati/Nat8l via epigenetic regulation, followed by determining the stress sensitivity by modulating serotonergic systems in the DRN. Focusing the region-specific BDNF regulation by Shati/Nat8l and modulating the DRN by Shati/Nat8l-BDNF pathway, we review the application of these findings as a novel therapeutic strategy for the depression in below.

## 4. Future Prospects

Finally, we discuss the prospects for a novel antidepressant treatment, from a clinical standpoint. BDNF has been reported to play an important role in depression pathogenesis in a region-specific manner. BDNF has both pro- and anti-depressant effects; therefore, the regulation of BDNF in the whole brain is not a beneficial strategy for the treatment of depression. As the regulation of BDNF by Shati/Nat8l is caused specifically in the dorsal striatum in response to stress, the downregulation of Shati/Nat8l would allow reduction of BDNF levels specifically in the dorsal striatum. Our series of studies demonstrated that the Shati/Nat8l-BDNF pathway in the dorsal striatum controls the DRN, which regulates the serotonergic system in the brain. Recently, R-ketamine has been used as an appealing approach for treating depression. A single administration of ketamine induced rapid and sustained antidepressant effects, even in patients with treatment resistance [162]. The effect of R-ketamine was mediated by the activation of serotoninergic systems in the DRN through the projection from the PFC via AMPA receptor stimulation in the PFC [163]. However, long-term treatment safety requires consideration as ketamine has psychological side effects such as dissociation, psychotomimetics, and abuse potential [164]. Directly targeting the DRN, which regulates serotonin in the whole brain, by Shati/Nat8l, showed a potent antidepressant effect similar to that of ketamine.

## 5. Conclusions

**Figure 2:**
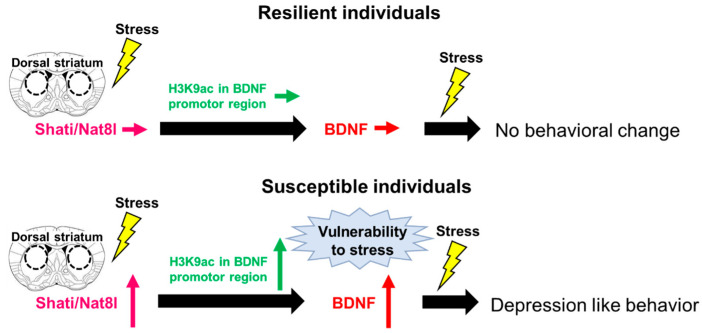
The Shati/Nat8l-BDNF pathway in the dorsal striatum determines stress sensitivity. A model summarizing the determination of stress sensitivity by the dorsal striatal Shati/Nat8l-BDNF pathway: resilient mice do not show the upregulation of Shati/Nat8l and BDNF expression by chronic social stress; thus, vulnerability to stress is not established and subsequent stress does not induce depression-like behaviors. Susceptible mice show upregulation of Shati/Nat8l and BDNF expression by chronic social stress, resulting in the establishment of a vulnerability to stress, in which subsequent stress contributes to depression-like behaviors.

The reinforcement of resilience to stress suppresses the progression of depression Reducing stress-induced damage may synergize with therapy for depression and accelerate the recovery. The results of our study demonstrate the pro-depressant function of dorsal striatal BDNF and its relationship to Shati/Nat8l in the biological mechanisms of stress-sensitivity determination (Figure 2). Decreased of the dorsal striatal BDNF-induced stress resilience via regulation of Shati/Nat8l may be a beneficial therapeutic strategy for depression. Taken together, the findings regarding Shati/Nat8l-BDNF pathway in the dorsal striatum have provided important insights into the molecular mechanisms underlying resilience and susceptibility in response to stress; thus, this could be a promising novel candidate as a therapeutic agent for depression by modulating sensitivity to stress.

## Figures and Tables

**Table 1:** List of studies on depression that investigated BDNF mRNA and/or protein function in the brain.

Brain region	Speceie	Trials	Effect to Depression Pathogenesis	Alteration of Expression Levels in Depression Individuals
PFC	Human	[69,70,71,72]	pro-depressant	increasing
	Animal	[76,77,78,79,80,81]		
Hippocampus	Human	[93,94,101,108]	pro-depressant	increasing
	Animal	[30,102,103,104,105,106,109]		
Mesolimbic pathway	Human	[111]	anti-depressant	decreasing
	Animal	[29,58,110,112,113,122]		
Dorsal striatum	Human	None	anti-depressant	decreasing
	Animal	[136]		
PFC: Prefrontal cortex				

## Data Availability

Data sharing not applicable.
